# Correction: Coronavirus endoribonuclease nsp15 suppresses host protein synthesis and evades PKR-eIF2α-mediated translation shutoff to ensure viral protein synthesis

**DOI:** 10.1371/journal.ppat.1014087

**Published:** 2026-03-31

**Authors:** Xiaoqian Gong, Shanhuan Feng, Jiehuang Wang, Bo Gao, Wenxiang Xue, Hongyan Chu, Shouguo Fang, Yanmei Yuan, Yuqiang Cheng, Min Liao, Yingjie Sun, Lei Tan, Cuiping Song, Xusheng Qiu, Chan Ding, Edwin Tijhaar, Maria Forlenza, Ying Liao

The images for Figs 8–10 are incorrectly switched. The image that appears as Fig 8 should be Fig 9, the image that appears as Fig 9 should be Fig 10, and the image that appears as Fig 10 should be Fig 8. The figure captions appear in the correct order. The authors have provided a corrected version of the figures here.

**Fig 8:**
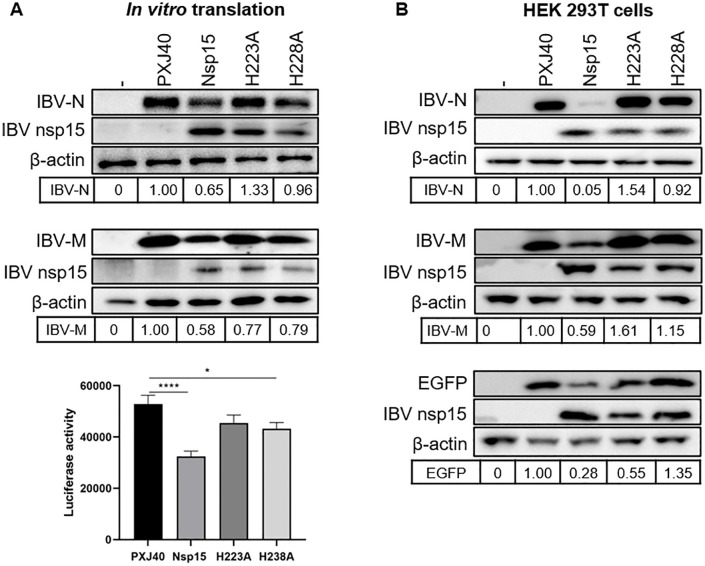
IBV nsp15 targets cytoplasmic factors to inhibit protein synthesis. **(A)** Plasmids encoding wild-type or catalytic-deficient IBV nsp15 and reporter plasmids encoding IBV N or IBV M, or luciferase DNA, were co-incubated with Rabbit Reticulocyte Lysate for 1 h, followed by Western blot analysis or luciferase assay. Density of the bands corresponding to the reporter proteins was normalized to the signal of β-actin and presented relative to the sample transfected with the empty vector PXJ40. ****:p < 0.0001; *:p < 0.05. **(B)** Plasmids encoding wild-type or catalytic-deficient IBV nsp15 and reporter plasmids encoding IBV N or IBV M, or EGFP, were co-transfected into HEK 293T cells for 24 h, followed by Western blot analysis. Band densities of IBV N, IBV M, or EGFP were quantified using ImageJ, normalized to the signal of β-actin, and presented relative to the PXJ40 group.

**Fig 9:**
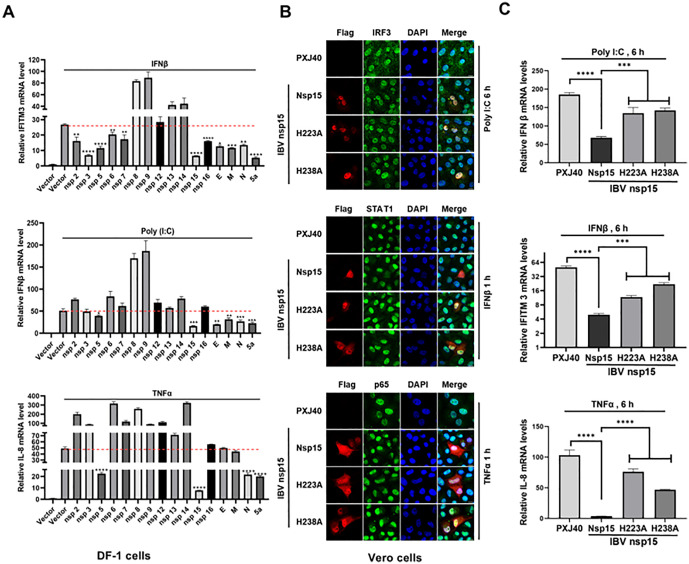
IBV nsp15 suppresses the transcriptional expression of antiviral genes IFN. β, IFITM3, and IL-8. **(A)** DF-1 cells were transfected with PXJ40 or plasmids encoding IBV proteins. At 24 h post-transfection, cells were either transfected with poly(I:C) or treated with IFNβ or TNFα, followed by real time qRT-PCR analysis to measure the transcription of IFNβ, IFITM3, and IL-8. **(B)** Vero cells were transfected with PXJ40 or plasmids encoding IBV nsp15, H223A, or H238A. At 24 h post-transfection, cells were either transfected with poly(I:C) or treated with IFNβ or TNFα. Immunofluorescence was performed to detect the nuclear translocation of IRF3, STAT1, and p65. Representative images are shown. Real time qRT-PCR was also conducted to measure the transcription of IFNβ, IFITM3, and IL-8. Statistical significance levels are denoted as follows: ns, P > 0.05; *P < 0.05; **P < 0.01; ***P < 0.001; ****P < 0.0001.

**Fig 10:**
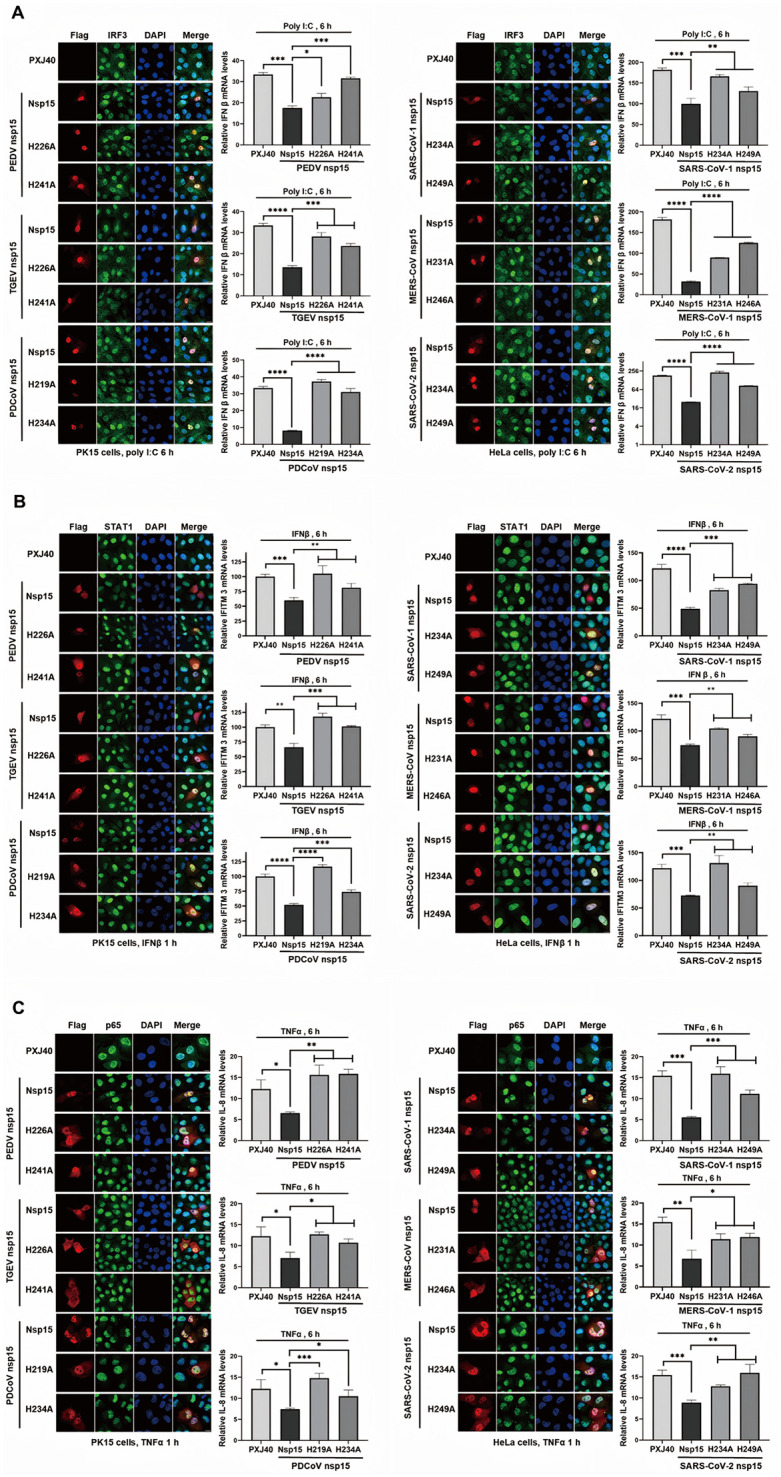
Coronavirus nsp15 suppresses the transcriptional expression of antiviral genes IFN. β, IFITM3, and IL-8. **(A-C)** PK15 or HeLa cells were transfected with PXJ40 or plasmids encoding nsp15 or catalytic-deficient mutants from porcine coronavirus (PEDV, TGEV, PDCoV) and human coronavirus (SARS-CoV-1, MERS-CoV, SARS-CoV-2). At 24 h post-transfection, cells were either transfected with poly(I:C) or treated with IFNβ or TNFα. Immunofluorescence staining was performed to detect the nuclear translocation of transcription factors IRF3, STAT1, and p65. Representative images are shown. Real time qRT-PCR was conducted to measure the transcription of IFNβ, IFITM3, and IL-8. Statistical significance levels are denoted as follows: ns, P > 0.05; *P < 0.05; **P < 0.01; ***P < 0.001; ****P < 0.0001.
