# Supplementary figures and images for: Coronavirus endoribonuclease nsp15 suppresses host protein synthesis and evades PKR-eIF2α-mediated translation shutoff to ensure viral protein synthesis
Source: PLoS Pathog. 2025 Mar 17;21(3):e1012987. doi: 10.1371/journal.ppat.1012987 (PMC11975131; doi:10.1371/journal.ppat.1012987)

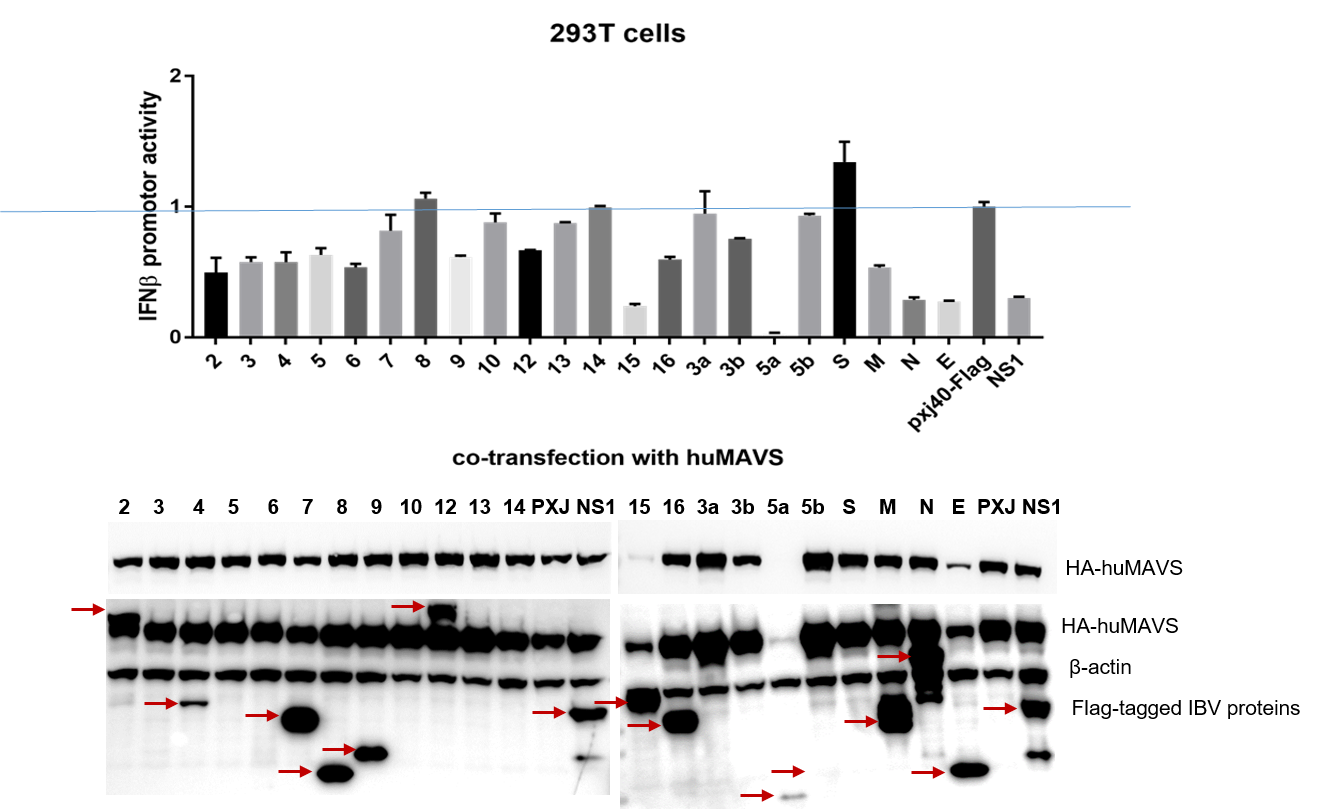

Supplement: S1 Fig — 293T cells were seeded in 96-well plates at a density of 1.5×10^4^ cells/well for the luciferase assay or in 12-well plates at a density of 1×10^5^ cells/well for Western blot analysis. Cells were co-transfected with plasmids encoding Flag-tagged IBV proteins (nsp2, nsp3, nsp4, nsp5, nsp6, nsp7, nsp8, nsp9, nsp10, nsp12, nsp13, nsp14, nsp15, nsp16, 3a, 3b, 5a, 5b, S, M, N, E), along with HA-huMAVS, the reporter plasmid encoding firefly luciferase driven by the inducible IFNβ promoter, and the control plasmid pRL-TK encoding Renilla luciferase driven by the constitutive HSV TK promoter. In parallel experiments, PXJ40 and a plasmid encoding Flag-tagged IAV NS1 were individually transfected as controls. After 24 h, cells in the 96-well plates were lysed, and the firefly and Renilla luciferase activities were measured. The activity of the IFNβ promoter was normalized to Renilla and presented relative to the PXJ40 control. The bars represent the average of two independently performed co-transfection experiments. Cells in the 12-well plates were lysed and subjected to Western blot analysis to verify protein expression. The membranes were first probed with an anti-HA antibody to detect huMAVS, followed by re-probing with an anti-Flag antibody to detect IBV proteins and IAV NS1 protein. Finally, the membranes were probed with an anti-actin antibody to detect actin as a loading control. The red arrows indicate the IBV protein bands. (TIF) [file ppat.1012987.s001.tif]

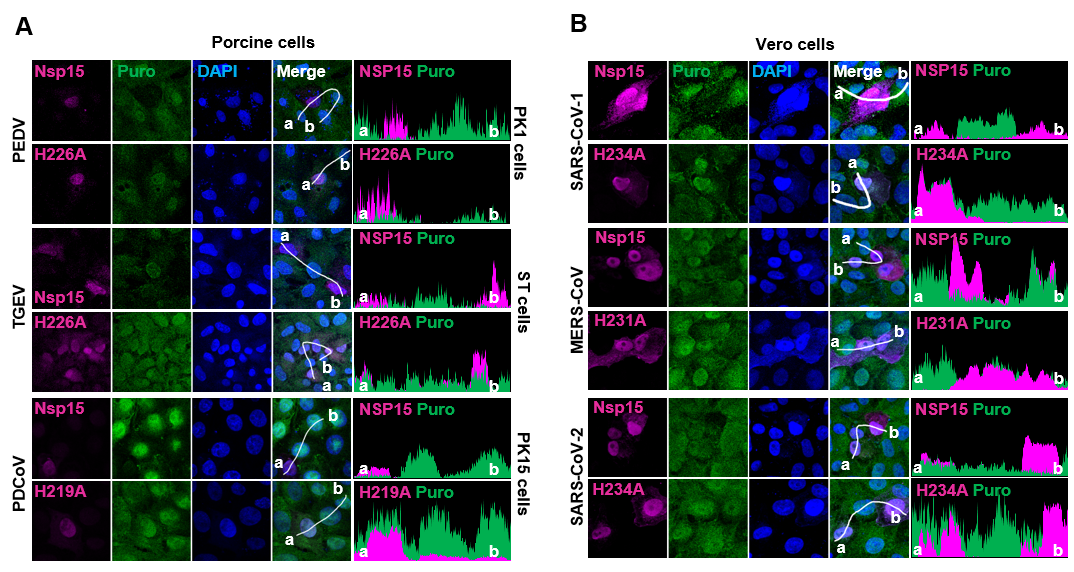

Supplement: S2 Fig — (A-B) Porcine cells (PK1, ST, and PK15) and Vero cells were transfected with wild-type nsp15 or the corresponding catalytic-deficient nsp15 from the indicated coronaviruses. At 23 h.p.t., cells were treated with puromycin (5 µg/mL) for 1 h. Indirect immunofluorescence staining was performed to detect nsp15 (magenta) and puromycin-labelled de novo synthesized peptides (green). Nuclei were labelled with DAPI (blue). Fluorescence intensity of nsp15 and puromycin signal along the white line (from a to b) is indicated by histogram plot. Representative images are shown. (TIF) [file ppat.1012987.s002.tif]

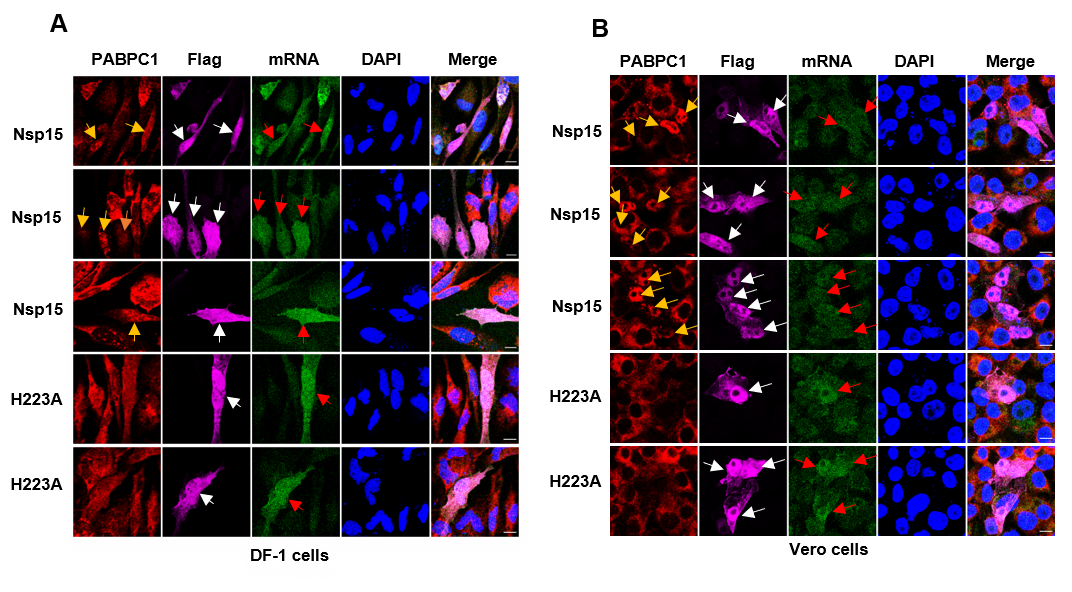

Supplement: S3 Fig — (A-B) Wild-type IBV nsp15 or the catalytic/oligomerization-deficient nsp15-H223A was transfected into DF-1 or Vero cells. After 24 h, in situ hybridization followed by indirect immunofluorescence was performed to visualize PABPC1 (red), nsp15 (magenta), and cellular mRNA (green). Nuclei were labelled with DAPI (blue). Representative images are shown. Yellow arrows indicate cells with nuclear accumulation of PABPC1. white arrows indicate cells expressing nsp15, and red arrows indicate the distribution of mRNA in cells expressing nsp15. Representative images are shown. (TIF) [file ppat.1012987.s003.tif]
