# Supplementary material for: Coronavirus endoribonuclease nsp15 suppresses host protein synthesis and evades PKR-eIF2α-mediated translation shutoff to ensure viral protein synthesis
Source: PLoS Pathog. 2025 Mar 17;21(3):e1012987. doi: 10.1371/journal.ppat.1012987 (PMC11975131; doi:10.1371/journal.ppat.1012987)
Supplement: S1 Table — (XLSX) [file ppat.1012987.s004.docx]

**S1 Table. Primer sequences used for real-time qPCR**

|  | **Primer Name** | **Sequence** |
| --- | --- | --- |
| 1 | ch-IFNβ Forward | 5'-AGCTCTCACCACCACCTTCTC-3' |
| 2 | ch-IFNβ Reverse | 5'-TGGCTGCTTGCTTCTTGTCCTT-3' |
| 3 | ch-IFITM3 Forward | 5'-TGGTGACGGTGGAGACG-3' |
| 4 | ch-IFITM3 Reverse | 5'-GGCAACCAGGGCGATGA-3' |
| 5 | ch-IL8 Forward | 5'-GCAGTTCTGGCTCTCCTCCTGGTTT-3' |
| 6 | ch-IL8 Reverse | 5'-GCTCGGTGTCAGCTTCACATCTTG-3' |
| 7 | ch-actin Forward | 5'-TATTGCTGCGCTCGTTGTTGAC-3' |
| 8 | ch-actin Reverse | 5'-GATACCTCTTTTGCTCTGGGCTTC-3' |
