# Supplementary material for: Coronavirus endoribonuclease nsp15 suppresses host protein synthesis and evades PKR-eIF2α-mediated translation shutoff to ensure viral protein synthesis
Source: PLoS Pathog. 2025 Mar 17;21(3):e1012987. doi: 10.1371/journal.ppat.1012987 (PMC11975131; doi:10.1371/journal.ppat.1012987)
Supplement: Attachment — Submitted filename: Response to Reviewers comments Nsp15 manuscript 20241127.docx [file ppat.1012987.s008.docx]

**Shanghai Veterinary Research Institute**

**Chinese Academy of Agricultural Sciences**

Nov 27, 2024

Editors-in-Chief

*Plos Pathogens*

Dear Editor,

I am writing to resubmit our manuscript entitled, “**Coronavirus endoribonuclease nsp15 induces host protein synthesis shutdown and ensures viral protein synthesis via evading PKR-eIF2****α-mediated anti-viral translation shutoff**”, for consideration of publication in *PLOS Pathogens*. We are grateful for the opportunity to revise our work in response to the constructive feedback provided by you and the reviewers. We have carefully considered all the comments and suggestions made by reviewers and have made the necessary revisions to improve the manuscript.

To make our conclusions more convincing, we conducted RIP-Seq RNA interactome and protein interactome analyses of nsp15 under viral infection conditions, supporting our conclusions. The results revealed that nsp15 binds to six viral RNAs, 257 host RNAs and 809 host proteins. GO and KEGG analyses showed that most of the host RNAs and proteins associated with nsp15 are involved in RNA splicing, processing, ribosome biogenesis, RNA binding, translation initiation, and the translation process. These findings support our conclusions and have been included in the manuscript.

Below are the responses to editors’ and reviewers’ comments and suggestion.

Your sincerely,

Ying Liao on behalf of the authors

Shanghai Veterinary Research Institute, Chinese Academy of Agricultural Sciences, China

Corresponding author:

Ying Liao, Phone: +86-21-34680291, Fax: +86-21-54081818,

Email: liaoying@ shvri.ac.cn

Dear Dr. Liao,

Thank you very much for submitting your manuscript "Coronavirus endoribonuclease nsp15 induces host protein synthesis shutdown and ensures viral protein synthesis via evading PKR-eIF2α-mediated anti-viral translation shutoff" for consideration at PLOS Pathogens. As with all papers reviewed by the journal, your manuscript was reviewed by members of the editorial board and by several independent reviewers. In light of the reviews (below this email), we would like to invite the resubmission of a significantly-revised version that takes into account the reviewers' comments.

Revisions should address the specific points made by each reviewer. We believe that these suggestions will help to improve your manuscript even further.

In particular, please pay careful attention to reviewer concerns regarding the following:

- Cytotoxicity analysis for nsp15 overexpression

**Response:** We analyze the cytotoxicity for nsp15 overexpression, there is no significant cytotoxicity when nsp15 is transient expressed within 26 h.

- Contradictory results when comparing the impact of nsp15 overexpression on exogenous and *de novo* protein synthesis inhibition

**Response:** The impact of nsp15 overexpression on exogenous and *de novo* protein synthesis inhibition is consistent.

- The role for endonuclease activity in changes in host gene expression or localization of cellular signaling molecules

**Response:** Nsp15 reduces the expression of IFNβ and IL-8, but does not inhibit the nuclear translocation of transcription factors IRF3 or p65. The endonuclease activity of Nsp15 is essential for the reduction of IFN-β and IL-8, suggesting that the endonuclease activity is responsible for the downregulation of these host gene expression. Since IRF3 and p65 can still translocate into the nucleus, the inhibition of IFN-β and IL-8 expression likely occurs post-transcriptionally.

- Contradictory results following infection and overexpression, including differences in localization of PABPC1 and the relevance of this protein in general

**Response:** During infection, both viral RNA and viral proteins are present. It has been reported that nsp15 associates with the replication/transcription complex and viral RNA. Consequently, nsp15 colocalizes with RdRp nsp12 during infection. During transfection, only nsp15 is expressed, resulting in a simpler scenario. In the absence of viral RNA and proteins to bind with nsp15, it is freely distributed in the nucleus and cytoplasm.

Regarding the localization of PABPC1, during wild-type IBV infection, the PKR-eIF2α-mediated translation shutoff does not occur. Viral mRNA binds to PABPC1, facilitating its translation and retaining PABPC1 in the cytoplasm. In contrast, during rIBV-nsp15-H238A infection, dsRNA accumulates due to the loss of nsp15’s function, triggering the PKR-eIF2α-mediated translation shutoff. This shuts off both host and viral mRNA translation, releasing PABPC1 and exposing its nuclear localization signal (NLS), resulting in its translocation to the nucleus.

During transfection, Nsp15 disrupts host protein translation, causing PABPC1 to dissociate from host mRNA and translocate to the nucleus. When nsp15 loses its endoribonuclease activity, it fails to inhibit host protein translation, allowing PABPC1 to remain in the cytoplasm, bound to host mRNA, and facilitating translation.

- The reproducibility and significance of protein synthesis inhibition as it relates to both the role of nsp15 endonuclease activity and the role of PKR.

**Response:** We conducted the experiment of knocking down PKR and infecting cells with IBV or rIBV-nsp15-H238A three times, and the results were consistently reproducible.

- Clarification of methods used to quantify puromycin labeling.

**Response:** The ImageJ software (NIH, USA) was utilized to quantify the intensities of bands in Western blot analyses, dot blot analyses for dsRNA, as well as the signal intensity of puromycin in immunofluorescence images. Additionally, Pearson’s correlation coefficient was calculated using ImageJ to analyze signal correlations in immunofluorescence images.

- Conclusions that may overstate the data

**Response:** We conducted RIP-Seq RNA interactome and protein interactome analyses of nsp15 under viral infection conditions, supporting our conclusions. The results revealed that nsp15 binds to six viral RNAs, 257 host RNAs and 809 host proteins. GO and KEGG analyses showed that most of the host RNAs and proteins associated with nsp15 are involved in RNA splicing, processing, ribosome biogenesis, RNA binding, translation initiation, and the translation process. These findings support our conclusions and have been included in the manuscript as supporting data.

We cannot make any decision about publication until we have seen the revised manuscript and your response to the reviewers' comments. Your revised manuscript is also likely to be sent to reviewers for further evaluation.

When you are ready to resubmit, please upload the following:

[1] A letter containing a detailed list of your responses to the review comments and a description of the changes you have made in the manuscript. Please note while forming your response, if your article is accepted, you may have the opportunity to make the peer review history publicly available. The record will include editor decision letters (with reviews) and your responses to reviewer comments. If eligible, we will contact you to opt in or out.

[2] Two versions of the revised manuscript: one with either highlights or tracked changes denoting where the text has been changed; the other a clean version (uploaded as the manuscript file).

Important additional instructions are given below your reviewer comments.

Please prepare and submit your revised manuscript within 60 days. If you anticipate any delay, please let us know the expected resubmission date by replying to this email. Please note that revised manuscripts received after the 60-day due date may require evaluation and peer review similar to newly submitted manuscripts.

Thank you again for your submission. We hope that our editorial process has been constructive so far, and we welcome your feedback at any time. Please don't hesitate to contact us if you have any questions or comments.

Sincerely,

Emily Ledgerwood, PhD
Guest Editor
PLOS Pathogens

Ashley St. John
Section Editor
PLOS Pathogens

Michael Malim
Editor-in-Chief
PLOS Pathogens
orcid.org/0000-0002-7699-2064
Reviewer's Responses to Questions

**Part I - Summary**
Please use this section to discuss strengths/weaknesses of study, novelty/significance, general execution and scholarship.

Reviewer #1: Non-structural proteins play an important role in the life cycle of viruses. These non-structural proteins play indispensable roles to suppress the physiological activity and immunity of host cells. In this paper, they studied showed the coronavirus endoribonuclease nsp15 inhibits host protein synthesis by targeting host RNA. This study revealed that nsp15 may regulate host protein expression by targeting host and viral RNA. While this result is somewhat convincing, a few concerns need to be addressed.

Reviewer #2: This resubmitted manuscript explores the role of CoV nsp15 in the induction of translational shutoff. While the role of nsp15 in reduction of viral dsRNA and prevention of PKR activation has previously been explored (Kinlder; Deng), the authors here show that this also holds true for another CoV, IBV. New findings, such as targets of nsp15 binding (including several RNA targets that regulated RNA expression) are novel, however some of the conclusions are overstated based on the data provided.

**Part II – Major Issues: Key Experiments Required for Acceptance**
Please use this section to detail the key new experiments or modifications of existing experiments that should be absolutely required to validate study conclusions.

Generally, there should be no more than 3 such required experiments or major modifications for a "Major Revision" recommendation. If more than 3 experiments are necessary to validate the study conclusions, then you are encouraged to recommend "Reject".

Reviewer #1:

1. Fig1 A, please explain why there are the low expression level of GFP in the absence of an inhibitor. I have another concern whether the expression of β-actin protein will be inhibited, since nsp15 inhibits host protein synthesis. This problem was common in the description that follows. Fig1 B, the authors needed to indicate whether the protein is cytotoxic, since nsp15 inhibits host protein synthesis.

**Response:** Thank you for your comments. Regarding Fig 1A, it demonstrates that the presence of nsp15 can suppress the expression of exogenously transfected plasmids encoding proteins. GFP serves as an exogenous reporter plasmid encoding protein, confirming that nsp15 not only inhibits the expression of MDA5, MAVS, and IRF7 but also broadly suppresses the expression of other plasmid encoding protein, including GFP. β-actin is an endogenous cytoskeletal protein that is already present in cells. Transient expression of nsp15 does not reduce the levels of β-actin that are already present. Therefore, the detected levels of β-actin remain consistent and uniform between the control group and the nsp15 expression group.

Figure 1B shows that the expression of nsp15 can inhibit endogenous protein synthesis. There is no significant cytotoxicity by transient expression of nsp15 within 26 h.

Fig. H1299 or Vero cells were transfected with nsp15 from diverse coronaviruses. At 24 h post-transfection, cells were treated with CCK8 for 2 h. Then, the OD value will be obtained at 450 nm by using the microplate reader. Data are presented as the means ± SD of three independent experiments. P values were calculated by Student`s test; ns, no significant difference.

2. Fig 4 showed some conflict with Fig 3. In the fig, the groups of MERS-CoV, SARS-CoV2 showed some ability of inhibition, however, the group of PDCoV showed weak ability of inhibition in protein synthesis. There are similar problems in S2 Fig.
   3. Fig8, although it has been reported in the previous studies, how to make out nsp15 inhibit nuclear translocation of IRF3 in this Fig.

**Response:**  Figure 3 shows that the suppression of exogenously expressed plasmids encoding proteins by the groups of MERS-CoV and SARS-CoV-2 nsp15 is not pronounced. Figure 4 and S2. Fig demonstrates that the inhibition of endogenous protein synthesis by nsp15 from MERS-CoV and SARS-CoV-2 is also weaker compared to nsp15 from other coronaviruses. The results from figures 3, figure 4, S2 are consistent. Regarding PDCoV nsp15, both Figures 3 and 4 demonstrate a strong inhibitory effect on protein translation.

Fig 3

Fig 4

S2 Fig

Regarding Figure 9 and Figure 10, although there are reports indicating that SARS-CoV-2 nsp15 inhibits the phosphorylation and nuclear translocation of IRF3 (<https://www.ncbi.nlm.nih.gov/pmc/articles/PMC10480782/>), we did not observe any inhibition of IRF3 nuclear translocation by coronavirus nsp15 in our experiments. Our interpret about Figure 9 is “As shown in Fig 9B, poly(I:C) stimulation resulted in the nuclear translocation of IRF3, while TNFα stimulation caused the nuclear translocation of p65. The wild type IBV nsp15 and the catalytic-deficient mutants H223A and H238A were unable to inhibit the nuclear translocation of IRF3 and p65, indicating these transcription factors can still initiate the transcription of downstream genes IFNβ and IL-8.” We include additional figures as evidence.

Reviewer #2: -

1. A number of times the authors state that differences in host gene expression and/or altered localization of signaling molecules (e.g. STAT) support the conclusion that nsp15 is targeting these host genes for degradation which is dependent on the endoU activity of nsp15. While this is a plausible hypothesis, this data alone is insufficient to support this. Alternative explanations could be that nsp15 is targeting other host RNAs that regulate expression of these proteins. This can be addressed by tempering these statements regarding what can be concluded from the data.

**Response:**  Thank you for your valuable feedback. We agree that our data suggest that nsp15 may target the antiviral mRNA for degradation, alternatively, it is also possible to target other host RNAs that regulate these transcripts. In the revised manuscript, we change the statement to: “Since IRF3 and p65 can still translocate into the nucleus, the inhibition of IFN-β and IL-8 expression likely occurs post-transcriptionally.” and “These results further suggest that coronavirus nsp15 targets IFNβ and IL-8 for degradation or, alternatively, targets other substrates to regulate the transcription levels of these antiviral genes.”

1. The conclusion that nsp15 is important for host translational shutoff independent of PKR is appealing but unsubstantiated by the data (specifically Fig 11D). Relative quantitation of the signal in PKR siRNA kd cells infected with WT or mutant virus show 0.74 and 0.89 relative change in translation respectively. It's unclear if this difference is biologically (or statistically) significant, and it is unclear whether this quantitation data is from a single experiment or from multiple experiments. Moreover, it appears that the relative difference in puromycin incorporation in WT vs mutant infected cells (without siRNA treatment) is different in 11D vs 11B, where 11B shows substantial reduction following infection with both viruses, but 11D shows significantly more reduction in the mutant infected cells vs WT. While biological variation in such assays may be expected, it's challenging to know how consistent and biologically significant the phenotype in 11D is.

**Response:** We repeated the PKR knockdown experiments three times and obtained consistent results. The mutant strain rIBV-nsp15-H238A showed a reduced inhibition of protein translation, with a partial recovery of protein synthesis in the absence of PKR, compared to PKR-positive cells. When comparing the inhibition of protein translation between wild-type IBV and the mutant strain rIBV-nsp15-H238A in PKR knockdown cells, rIBV-nsp15-H238A exhibited less inhibition, suggesting that the loss of nsp15 activity partially alleviated the inhibition of protein translation. The results were consistent across three independent experiments and showed statistical significance.

Additionally, in Figures 11B and 11D, Figure 11B shows a substantial reduction following infection with both viruses, while Figure 11D shows a significantly greater reduction in the mutant-infected cells compared to the wild type. This difference is due to the sampling time in Figure 11D, which was 18 hours post-infection. We adjusted the sampling time in the 11D experiment to 24 hours post-infection, at which point the inhibition of protein translation by wild-type IBV and the mutant strain in PKR-positive cells showed a consistent pattern. We replaced Figure 11D with new results. The new results of additional experiments were presented as follows.

**Part III – Minor Issues: Editorial and Data Presentation Modifications**
Please use this section for editorial suggestions as well as relatively minor modifications of existing data that would enhance clarity.

Reviewer #1:

1. S1 Fig, In the nsp15 expression group, why β-actin expressed in high level. And why some IBV protein not detected.

Reply: In S1 Fig, β-actin is a host cell cytoskeletal protein that is stably present in cells, which is different from the transiently expressing of HA-huMAVS encoded by plasmid. The expression of HA-huMAVS is suppressed by nsp15, whereas the level of endogenous actin in the cells is not affected by nsp15. Thus, the β-actin is present in steady and high level in the nsp15 expression group.

There are differences in the expression levels of IBV proteins, with some viral proteins expressed at lower levels and difficult to detect. We marked the detectable IBV proteins with red arrows in S1 Fig, among which both nsp15 and 5a are expressed and clearly inhibit the expression of the co-transfected plasmid encoding HA-huMAVS.

2. Fig2 A, why are NSP15 not detected in NSP15 transfection group. This description of the wild-type nsp15 is inaccurate, because there are only transfection by NSP15 plasmid, but infection by virus.

**Response:**  Fig2 A, Nsp15 inhibits protein synthesis, which could also lead to its own synthesis being suppressed even in transfection condition. This could explain why the expression level of nsp15 is lower than that of the mutant variants H223A and H238A, which have lost the ability to inhibit protein synthesis. Regarding D285A and D315A, they have lost the ability to form the correct hexamer. Although they may lose the ability to suppress reporter plasmid expression, the inability of D285A and D315A to form hexamers could result in instability and increased degradation, which is reflected in their lower expression levels.

Thank you for pointing out that the naming of wild-type nsp15 was inappropriate. We named the unmutated nsp15 as wild-type nsp15 to distinguish it from the mutant forms of nsp15. After consideration, we have decided to retain the name wild-type nsp15.

3. Is the wild-type nsp15 transfection or viral infection? If it’s transfection, why the expression level was weak?

**Response:**  Nsp15 inhibits protein synthesis, which could also lead to its own synthesis being suppressed in transfection condition.

Figures 1 to 10 show the results of nsp15 plasmid transfection and its effect on inhibiting protein translation, while Figures 11, 12, and 13 display the regulatory effects of IBV infection on protein translation, as well as the role of nsp15 in inhibiting protein translation and its subcellular localization during viral infection.

4. Fig. 5B, the nuclear size varied greatly among the groups. Authors need to unify the scales.

**Response:**  Thank you for your suggestion regarding Figure 5B. We unify the scales for the nuclear size across the images in the revised manuscript to ensure consistency and clarity in the presentation of our data.

5. Fig. 6, these viral nsp15 should indicated in the fig, rather than just the virus name, which can be misleading. And it's hard to make out the difference between these groups.

**Response:**  We agree that clearly indicating the specific viral nsp15 proteins rather than just the virus names will improve clarity. In the revised manuscript, we update the labels in figures to ensure that the differences between the groups are more easily discernible.

6. Fig11A, the labeling of these symbols (+,-) is confusing.

**Response:**  In Figure 11, due to the need to represent two types of viral infections and several time points, we have used "+" to indicate the presence of the relevant virus infection and "-" to indicate its absence. We believe this representation helps to save space in the figure.

Reviewer #2: n/a

PLOS authors have the option to publish the peer review history of their article ([what does this mean?](https://c05y1x9s.r.us-east-2.awstrack.me/L0/https:%2F%2Fjournals.plos.org%2Fplospathogens%2Fs%2Feditorial-and-peer-review-process%23loc-peer-review-history/1/010f01926a2a124d-c6010925-2b90-457e-bb26-cbec1c2a4644-000000/xZv6lirbQur7kXHUJfh8DiP-lOA=179)). If published, this will include your full peer review and any attached files.

If you choose “no”, your identity will remain anonymous but your review may still be made public.

**Do you want your identity to be public for this peer review?** For information about this choice, including consent withdrawal, please see our [Privacy Policy](https://c05y1x9s.r.us-east-2.awstrack.me/L0/https:%2F%2Fwww.plos.org%2Fprivacy-policy/1/010f01926a2a124d-c6010925-2b90-457e-bb26-cbec1c2a4644-000000/84QcsepIOciKUn5Su8M7Wp_Q89E=179).

Reviewer #1: No

Reviewer #2: No

Figure Files:

While revising your submission, please upload your figure files to the Preflight Analysis and Conversion Engine (PACE) digital diagnostic tool, [https://pacev2.apexcovantage.com](https://c05y1x9s.r.us-east-2.awstrack.me/L0/https:%2F%2Fpacev2.apexcovantage.com%2F/1/010f01926a2a124d-c6010925-2b90-457e-bb26-cbec1c2a4644-000000/m5p377rFm-Ela2l-oon_v3yBhes=179). PACE helps ensure that figures meet PLOS requirements. To use PACE, you must first register as a user. Then, login and navigate to the UPLOAD tab, where you will find detailed instructions on how to use the tool. If you encounter any issues or have any questions when using PACE, please email us at [figures@plos.org](mailto:figures@plos.org).

Data Requirements:

Please note that, as a condition of publication, PLOS' data policy requires that you make available all data used to draw the conclusions outlined in your manuscript. Data must be deposited in an appropriate repository, included within the body of the manuscript, or uploaded as supporting information. This includes all numerical values that were used to generate graphs, histograms etc. For an example see here on PLOS Biology: http://www.plosbiology.org/article/info%3Adoi%2F10.1371%2Fjournal.pbio.1001908#s5.

Reproducibility:

To enhance the reproducibility of your results, we recommend that you deposit your laboratory protocols in protocols.io, where a protocol can be assigned its own identifier (DOI) such that it can be cited independently in the future. Additionally, PLOS ONE offers an option to publish peer-reviewed clinical study protocols. Read more information on sharing protocols at [https://plos.org/protocols?utm_medium=editorial-email&utm_source=authorletters&utm_campaign=protocols](https://c05y1x9s.r.us-east-2.awstrack.me/L0/https:%2F%2Fplos.org%2Fprotocols%3Futm_medium=editorial-email%26utm_source=authorletters%26utm_campaign=protocols/1/010f01926a2a124d-c6010925-2b90-457e-bb26-cbec1c2a4644-000000/LPUc9Rb0rfJ3QmG1_kDiMETupp4=179)
