# Supplementary material for: Coronavirus endoribonuclease nsp15 suppresses host protein synthesis and evades PKR-eIF2α-mediated translation shutoff to ensure viral protein synthesis
Source: PLoS Pathog. 2025 Mar 17;21(3):e1012987. doi: 10.1371/journal.ppat.1012987 (PMC11975131; doi:10.1371/journal.ppat.1012987)
Supplement: Attachment — Submitted filename: Response letter to reviewers 20240819.docx [file ppat.1012987.s007.docx]

**Shanghai Veterinary Research Institute**

**Chinese Academy of Agricultural Sciences**

August 21, 2024

Editors-in-Chief

*Plos Pathogens*

Dear Editors,

On behalf of my co-authors, we are re-submitting the original research article “**Corona****virus endoribonuclease nsp15 induces host protein synthesis shutdown and ensures viral protein synthesis via evading PKR-eIF2-mediated anti-viral translation shutoff**” for consideration of publication in *Plos Pathogens.* All co-authors have seen and agreed with the contents of the manuscript and there is no financial interest to report. We certify that the submission is original work and is not under review at any other publication.

This study reveals a novel mechanism by which the endoribonuclease nsp15, encoded by coronaviruses, suppresses protein synthesis. We found that Infectious Bronchitis Virus (IBV) nsp15 is associated with host translation shutdown and triggers PABPC1 nuclear relocation by targeting cytoplasmic factors, with its endoribonuclease activity being indispensable. We also examined the inhibitory function on protein translation and PABPC1 nuclear relocation of nsp15 from PEDV, TGEV, PDCoV, SARS-CoV-1, MERS-CoV, and SARS-CoV-2. The data from PEDV, TGEV, PDCoV, and SARS-CoV-1 demonstrate that the protein translation inhibitory function of nsp15 is conserved among these coronaviruses, while nsp15 from MERS-CoV and SARS-CoV-2 exhibits a lesser effect.

Furthermore, we determined that wild-type IBV infection inhibits host protein translation via a PKR-eIF2α-independent mechanism, with PABPC1 remaining in the cytoplasm, likely binding to viral mRNA for translation. Intriguingly, the nsp15 catalytic null mutant rIBV-nsp15-H238A also suppresses protein synthesis via the PKR-eIF2α pathway, accompanied by viral RNA accumulation, PKR activation, PABPC1 nuclear relocation, and reduced viral protein levels. rIBV-nsp15-H238A loses its ability to shut down protein translation in PKR knockdown cells, suggesting that functional nsp15 plays a crucial role in helping the virus shut down host protein synthesis in a PKR-eIF2α-independent manner. The PKR-eIF2α mediated translation shut off is detrimental to viral replication, as evidenced by the reduced viral protein production. Therefore, we conclude that nsp15 is not only responsible for reducing viral dsRNA, thereby avoiding the activation of PKR-eIF2α, a key checkpoint of host-mediated translation initiation shutoff that is detrimental to both host and viral protein synthesis, but also targets cellular RNA to specifically inhibit host protein translation (As evidenced by RIP experiments and qRT-PCR).

We believe that our study makes a significant contribution to the literature by elucidating the mechanism of host protein translation shutoff induced by coronavirus infection and highlighting the novel function of the endoribonuclease nsp15, which was previously unknown. Therefore, this study provides new targets for the development of drugs or vaccines to control the spread of coronaviruses. As the endoribonuclease is a conserved genetic marker in Nidovirales (nsp15 in coronavirus and nsp11 in arterivirus), this finding represents a breakthrough in understanding the regulation of host translation machinery by this order of viruses.

Regarding the emergence of new coronaviruses and their serious threat to public safety and animal health, we believe that this study will be of significant interest to the readers. I sincerely hope that our work will be considered suitable for publication in PLOS Pathogens and look forward to hearing from you in the near future.

Your sincerely,

Ying Liao and Maria Forlenza on behalf of the authors

Shanghai Veterinary Research Institute, Chinese Academy of Agricultural Sciences, China

Corresponding authors:

Ying Liao and Maria Forlenza, Phone: +86-21-34680291, Fax: +86-21-54081818,

Email: liaoying@ shvri.ac.cn, maria.forlenza@wur.nl

We have carefully considered all the comments and suggestions made by reviewers and have made the necessary revisions to improve the manuscript. Below are the responses to editors’ and reviewers’ comments and suggestion.

Reviewer #1: In this manuscript the authors look at the effects of overexpression of viral Nsp15 in different mammalian cell lines. They demonstrate that co-expression of Nsp15 with other genes such as MAVS and GFP leads to a decrease in their translation as compared to other viral proteins or catalytically inactive variants of Nsp15. Next the authors used a puromycin assay to show that over-expression of Nsp15 leads to a decrease in protein synthesis. They also demonstrate that Nsp15 overexpression alters the localization of PABPC1. Finally, the authors assessed translation and PABPC1 localization in IBV-WT and IBV-Nsp115-H238A virus infected cells. They found that PABPC1 accumulates in the nucleus with the H238A mutant but not WT-virus – opposite of the effects observed with the overexpression experiments. The authors suggest that this difference is in-part due to the altered cellular localization of Nsp15 in the context of over-expression versus virus infection. This experiment highlights the major flaw of this manuscript, over-expression experiments with Nsp15 do not mimic what is seen in virus infected cells. Nsp15 is a largely non-specific nuclease, thus it is the opinion of this reviewer that overexpression of Nsp15 in mammalian cells is likely toxic. It has previously been shown that over-expression of SARS1 WT Nsp15 in E. coli is toxic to bacteria (Guarino et al JMB 2005). Moreover, it is well established that overexpression of toxic proteins is known to lead to proteotoxic stress effects such as the shutdown of translation and the nuclear accumulation of PABPC1. Therefore, the observations made in this paper are likely general effects of overexpression of a toxic protein and not specific to Nsp15. For the authors to conclude that Nsp15 plays a role in shutting off host protein synthesis they would need to provide more concrete evidence from experiments carried out with virus infected cells. The few experiment that address this in the manuscript are not sufficient to make this claim.

**Response:** Thank you for your thoughtful and constructive feedback. We have carefully considered your comments and have conducted additional experiments to strengthen our conclusions. Below, we outline the new data and clarifications provided in the revised manuscript:

1. **New Experimental Evidence:**
   (1) **RNA Immunoprecipitation (RIP):** We conducted RIP experiments that demonstrate nsp15 binds to both host RNA and viral RNA during infection. This supports our hypothesis that nsp15 targets host RNA to inhibit protein synthesis.
   (2) **Inhibition of Antiviral Gene Transcription:** We found that nsp15 inhibits the transcription of several antiviral genes, including *IFNβ*, *IFITM3*, and *IL-8*. This inhibitory effect is likely mediated through RNA cleavage, as evidenced by the catalytic-deficient nsp15 mutant, which loses its ability to inhibit these genes.
   (3) **Northern Blot Analysis:** We used Northern blotting to show that nsp15 targets viral RNA for cleavage. In cells infected with the catalytic-deficient rIBV-nsp15-H238A mutant, viral RNA accumulates, further confirming nsp15's role in viral RNA reduction. By this way, nsp15 helps the virus evade the shutdown of protein synthesis caused by the PKR-eIF2α pathway, which is detrimental to viral protein translation.
   (4) **PKR Knockdown Experiments:** In PKR knockdown cells, the catalytic-deficient rIBV-nsp15-H238A mutant loses its ability to inhibit protein translation, demonstrating that nsp15 plays a key role in targeting host RNA to inhibit translation, independent of the PKR-eIF2α pathway.
2. **Clarification on Overexpression Artifacts:**
   (1) We acknowledge the potential discrepancies between nsp15 overexpression experiments and viral infection contexts. While it is known that overexpression of toxic proteins like SARS-CoV-1 WT nsp15 can lead to proteotoxic stress effects, we provide a novel finding that coronavirus nsp15 specifically targets host factors to shut down host protein synthesis.
   (2) In the revised manuscript, we explain why overexpression studies might not fully mimic the viral infection environment but emphasize that our findings from virus-infected cells (including RIP, Northern blot, and PKR knockdown experiments) provide robust evidence for nsp15’s role in host protein synthesis inhibition during infection.

We believe that these new experiments and clarifications significantly strengthen the manuscript. These findings indicate that Nsp15 plays dual roles during in regulating translation by inhibiting host protein synthesis during infection and aiding the virus in evading the protein synthesis shutdown mediated by the PKR-eIF2α pathway, which would otherwise hinder viral protein translation. This new discovery enhances the functional understanding of Nsp15.

We hope these revisions address your concerns and provide the necessary evidence to support our conclusions. We look forward to your feedback on our revised manuscript.

Reviewer #2: The authors of this article have previously implicated the endoribonuclease nsp15 of coronaviruses (nsp15 EndoU) in the evasion of host innate antiviral response by decreasing the amount of viral dsRNA (ref. 29). Here, they focused on the potential role of nsp15 EndoU in inhibition of host cell protein synthesis. They showed that overexpression of catalytically active nsp15 of the infectious bronchitis virus (IBV) and some other coronaviruses inhibits cellular protein synthesis and results in the nuclear retention of PABPC1. In addition, their FISH/oligo-dT probe analysis opened the possibility that at least in some cells nsp15 targets host mRNAs. They found that rabbit reticulocyte lysates recapitulate the nsp15-mediated translational inhibition and further hypothesized that nsp15 acts to displace PABPC1 from its complex with mRNAs. Unfortunately, no data were provided in support of this hypothesis. It should be noted that in infected cells, the function of nsp15 might be regulated through interaction with other viral proteins. Moreover, after overexpression, the levels of nsp15 would be expected to be much higher than in infected cells. Also, as shown here, nsp15 exhibits different subcellular localization after plasmid transfection and IBV infection. These considerations cast doubt on the physiological significance of the above mentioned results.

**Response:** In the revised manuscript, we have included additional experiments to strengthen our conclusion that nsp15 targets host RNA. Specifically:

1. **RNA Immunoprecipitation (RIP):** We conducted RIP experiments that demonstrate nsp15 binds to both host RNA and viral mRNA during infection. This finding supports our hypothesis that nsp15 directly interacts with host RNA, contributing to the inhibition of protein synthesis.
2. **Antiviral Gene Transcription Inhibition:** We observed that overexpressed nsp15 inhibits the transcription of several antiviral genes, including IFNβ, IFITM3, and IL-8, likely through RNA cleavage. This conclusion is supported by the fact that a catalytically deficient nsp15 mutant loses its ability to inhibit these genes, underscoring the importance of its nuclease activity.
3. **Northern Blot Analysis:** Northern blot experiments further confirmed that nsp15 targets viral RNA for cleavage, as evidenced by the accumulation of viral RNA in cells infected with the catalytically deficient rIBV-nsp15-H238A mutant. This finding highlights nsp15's role in viral RNA reduction, facilitating the virus's evasion of the PKR-eIF2α pathway, which is detrimental to viral protein translation.
4. **PKR knockdown experiments:** in PKR knockdown cells, the catalytic deficient rIBV-nsp15-H238A loses its capability to inhibit protein translation, further demonstrating that nsp15 is involved in targeting host RNA to inhibit protein synthesis.

In addition, we explain the relationship between nsp15 activity and the nuclear translocation of PABPC1. We hypothesize that nsp15 binding to host RNA leads to PABPC1's nuclear translocation. Previous studies suggest that a reduction in cytoplasmic RNA exposes PABPC1’s nuclear localization signal. Our experiments using an in vitro translation system confirmed that, in a nucleus-free rabbit reticulocyte lysate system, PABPC1 does not translocate to the nucleus, yet nsp15 still inhibits the expression of co-incubated plasmids. This finding indicates that PABPC1 nuclear translocation is not a prerequisite for nsp15-mediated translation inhibition. Instead, it suggests that nsp15 inhibits protein synthesis, causing PABPC1 to be released from host mRNA and subsequently translocate to the nucleus. The nuclease activity of Nsp15 is involved in both translation inhibition and promoting PABPC1 nuclear translocation, further suggesting that it likely targets host RNA for cleavage, resulting in translation shutdown and PABPC1 nuclear translocation. In cells infected with wild-type IBV, translation shutdown occurs without PABPC1 translocation into the nucleus, implying that viral RNA binds to PABPC1, retaining it in the cytoplasm. However, in cells expressing only nsp15, where viral RNA is absent, PABPC1 translocates to the nucleus upon inhibition of host cell RNA by nsp15. When rIBV-nsp15-H238A, which lacks nuclease activity, is present, it activates the PKR-eIF2α pathway, leading to a global shutdown of translation for both host and viral RNA, resulting in PABPC1 nuclear translocation. This observation contrasts with the effects seen in cells infected with wild-type IBV or those overexpressing the nsp15 nuclease-deficient protein.

Through RIP experiments using a nsp15 monoclonal antibody, we confirmed that during viral infection, nsp15 interacts with both viral and host RNA. Recent reports indicate that double membrane vesicles (DMVs) formed during coronavirus infection have pores for material transport in and out, further suggesting that although Nsp15 is primarily located in the replication-transcription complex, it may shuttle inside and outside the DMV, interacting with ribosomes and host RNA outside the DMV. We are the first to discover nsp15’s role in inhibiting host protein translation and to explore this conserved function and mechanism of action. While this study cannot answer all scientific questions, it serves as a stepping stone, leaving some questions to be addressed in future research.

We acknowledge that viral infection is more complex than viral protein plasmid transfection, and careful analysis of these differences is essential. The biological phenomena we observe are the results of multifaceted interactions between the virus and host cells. All research findings represent a small part of the whole picture, and accurately reporting these phenomena while striving to analyze their underlying mechanisms contributes significantly to the scientific field. As for the parts that remain unexplained, we can continue to explore and investigate them further. We have presented these phenomena transparently and have made our best efforts to analyze the underlying reasons, demonstrating our commitment to scientific rigor. We believe that this openness should be encouraged, as it fosters further exploration and understanding of complex biological processes.

Reviewer #3: Building upon their previous work (citation # 29 / PMID: 33635931), Gong and colleagues report that CoV nsp15 induces host protein synthesis shutoff when over-expressed in isolation in vertebrate cell lines. Mechanistically, they find that nsp15 mutations that disable oligomerization or catalytic activity can no longer inhibit host protein synthesis; however, they do not detect host mRNA degradation nor other RNA targets with the methods employed. Host protein synthesis shutoff phenotypes were evident for nsp15 from some coronaviruses (PEDV, TGEV, PDCoV, and SARS-CoV-1) but not others (MERS-CoV and SARS-CoV-2). PABPC1 relocalized from the cytoplasm to the nucleus when host protein synthesis shutoff phenotypes were evident. When over-expressed in isolation, by itself, nsp15 subcellular localization was more diffuse than that observed during infection by Infectious Bronchitis Virus (IBV). Polysome gradients, in conjunction with Western blots, were used to examine whether nsp15 cofractionates with ribosomes and/or polysomes. Finally, using wildtype and nsp15 mutant Infectious Bronchitis Virus (IBV), the authors examine host protein synthesis shutoff and PABPC1 subcellular localization in infected cells. In a wide-ranging working model (Fig. 12), the authors conclude that nsp15 induces host protein synthesis shutoff (when over-expressed in isolation), via mechanisms that require catalytic activity but no evident mRNA degradation; and that host protein synthesis shutoff occurs by distinct mechanisms in cells infected with either wildtype or nsp15 mutant Infectious Bronchitis Virus (IBV), via undefined host pathways.

Critique. This is a wide-ranging investigation that has some merit; however, the non-physiologic over-expression of nsp15, among other notable weaknesses noted below, undermine the meritorious aspects of the study. Some experimental data is of high quality; however, other experimental data is of low quality. The author’s conclusion that CoV endoribonuclease nsp15 induces host cellular protein synthesis shutoff is likely not correct under physiologic conditions.

**Response:** We appreciate the reviewer's insightful comments and concerns. In response, we have conducted additional experiments and made significant revisions to the manuscript to clarify and strengthen our findings regarding nsp15’s role in targeting host mRNA.

**New Experimental Evidence:** In the revised manuscript, we present additional data to support the conclusion that nsp15 targets host mRNA:

1. **RNA Immunoprecipitation (RIP):** We performed RIP experiments demonstrating that nsp15 binds to both host and viral RNA during infection. This finding underscores the direct interaction of nsp15 with host RNAs, suggesting its involvement in regulating host gene expression.
2. **Inhibition of Antiviral Gene Transcription:** We observed that nsp15 inhibits the transcription of several key antiviral genes. This inhibition is likely due to RNA cleavage, as the catalytically inactive nsp15 variant lost its ability to inhibit transcription. This result further supports the role of nsp15's EndoU activity in modulating host responses.
3. **Northern Blot Analysis:** We utilized Northern blotting to show that nsp15 directly cleaves viral RNA. In cells infected with the catalytic-deficient rIBV-nsp15-H238A mutant, we detected an accumulation of viral RNA, which confirms nsp15's role in reducing viral RNA levels and emphasizes its significance in viral replication and host interaction.
4. **PKR Knockdown Experiments:** In cells with PKR knocked down, the catalytic-deficient rIBV-nsp15-H238A mutant lost its ability to inhibit protein translation. This observation further demonstrates that nsp15's role in inhibiting protein synthesis involves targeting host RNA, likely as a mechanism to circumvent the PKR-eIF2α pathway.

**Manuscript Revisions:** We have revised the manuscript to more clearly present these results and their implications. The new data provide a more comprehensive understanding of how nsp15 contributes to host protein synthesis shutoff and the viral life cycle.

Major points

1. Non-physiologic conditions. CoV nsp15 only induces host protein synthesis shutoff when over-expressed in isolation in vertebrate cell lines under non-physiologic conditions. The biological significance of such observations is highly questionable.

**Response:** We understand the reviewer's concern regarding the non-physiologic conditions in our overexpression experiments. To address this, we conducted experiments under more physiologically relevant conditions by focusing on the role of nsp15 during actual viral infection.

In our revised manuscript, we present data from PKR knockdown experiments. These experiments are crucial because they allow us to rule out the PKR-eIF2α signaling pathway, which is typically activated by the accumulation of viral double-stranded RNA during infection with the catalytic-deficient rIBV-nsp15-H238A. By knocking down PKR expression, we were able to isolate the effect of nsp15 on protein translation independent of this pathway.

- In PKR knockdown cells, wild-type IBV continued to inhibit protein translation effectively.
- However, the catalytic-deficient rIBV-nsp15-H238A lost its ability to inhibit protein translation in these cells.

These results demonstrate that nsp15's ability to inhibit protein translation is not merely an artifact of overexpression under non-physiologic conditions. Instead, nsp15 actively targets host RNA during viral infection, contributing to the inhibition of host protein synthesis. This underscores the biological relevance of nsp15's function in the viral life cycle and its role in host-virus interactions.

2. Weaknesses in experimental approach fail to detect host RNA targets of nsp15 catalytic activity. CoV nsp15 mutations that disable oligomerization or catalytic activity can no longer inhibit host protein synthesis under the conditions of the experiment; however, host RNA targets of nsp15 catalytic activity are not detected by the experimental approaches used in the study. This weakness in the experimental dataset leads to speculation as noted below.

**Response:** We acknowledge the reviewer's concern regarding the detection of host RNA targets of nsp15. To address this, we have included additional experiments in the revised manuscript to provide more direct evidence that nsp15 targets host RNA.

1. **RNA Immunoprecipitation (RIP) Experiments:** We performed RIP experiments using a specific antibody against nsp15, which allowed us to demonstrate that nsp15 binds to both host and viral RNA during viral infection. This experiment provides direct evidence that nsp15 interacts with host RNA, supporting our hypothesis that it plays a role in host RNA cleavage.
2. **Inhibition of Antiviral Gene Transcription:** We observed that overexpressed nsp15 inhibits the transcription of several antiviral genes, including IFNβ, IFITM3, and IL-8. Importantly, this inhibitory effect was absent in the catalytic-deficient nsp15, strongly suggesting that the observed inhibition is due to the cleavage of host RNA by nsp15.

These additional experiments significantly strengthen the dataset by providing concrete evidence that nsp15 targets host RNA during viral infection. This reduces the speculative nature of our conclusions and supports the physiological relevance of nsp15's catalytic activity in inhibiting host protein synthesis.

3. Fanciful interpretations & speculation. The authors speculate that nsp15 can bind to host mRNAs, without degrading them, displacing PABP1 from host mRNA, provoking its relocalization to the nucleus, thereby inducing host protein synthesis shutoff.

**Response:** We appreciate the reviewer's feedback and understand the concern regarding the speculative nature of our initial interpretation. To address this, we have conducted additional experiments and revised our manuscript to provide more concrete evidence supporting our hypothesis.

1. **PABPC1 Nuclear Translocation:** In cells expressing Nsp15, we observed a decrease in protein synthesis levels accompanied by the nuclear translocation of PABPC1. Based on previous reports suggesting that a reduction in cytoplasmic RNA can expose the nuclear localization signal of PABPC1, we hypothesize that Nsp15 binds to host RNA, leading to this translocation. This is consistent with findings in the literature that relate cytoplasmic RNA levels to PABPC1 localization.
2. **In Vitro Translation System Experiments:** Using a nucleus-free rabbit reticulocyte lysate system, we confirmed that PABPC1 does not translocate to the nucleus in the absence of a nucleus, yet Nsp15 still inhibits the expression of co-incubated plasmids. This suggests that PABPC1 nuclear translocation is not a prerequisite for Nsp15-mediated translation inhibition. Instead, it appears that Nsp15's nuclease activity is responsible for both translation inhibition and promoting PABPC1 nuclear translocation, likely by targeting host RNA for cleavage.
3. **RNA Immunoprecipitation (RIP) and RT-PCR Results:** RIP experiments confirmed that Nsp15 binds to host RNA. Furthermore, RT-PCR results showed that Nsp15 reduced host mRNA level such as IFNβ, ISG IFITM3, IL-8. This provides direct evidence that Nsp15’s interaction with host RNA is not merely speculative but is indeed functionally significant, leading to translation shutdown and PABPC1 nuclear translocation.
4. **PABPC1 Nuclear Translocation during IBV and rIBV-nsp15-H238A infection:** In cells infected with wild-type IBV, translation shutdown occurs without PABPC1 translocation into the nucleus, implying that viral RNA binds to PABPC1, retaining it in the cytoplasm. However, in cells expressing only nsp15, where viral RNA is absent, PABPC1 translocates to the nucleus upon inhibition of host cell RNA by nsp15. When rIBV-nsp15-H238A, which lacks nuclease activity, is present, it activates the PKR-eIF2α pathway, leading to a global shutdown of translation for both host and viral RNA, resulting in PABPC1 nuclear translocation.

These findings reduce the speculative nature of our original interpretation and provide a more robust basis for our conclusions regarding the role of Nsp15 in host protein synthesis shutoff.

4. Polysome data (Fig. 11) is low quality – does not support authors interpretations. Nsp15 is detected at different molecular weights from one panel to the next in the Western blot. These are not convincing data of nsp15 in either panel. Specificity of antibodies is not established in these Western blot panels. One cannot conclude from these data that nsp15 co-fractionates with host polysomes.

**Response:** We appreciate the reviewer's detailed critique of the polysome data. We have carefully reviewed the results and made the following adjustments to address the concerns:

1. **Specificity of nsp15 Antibody:** The antibody used against nsp15 is a monoclonal antibody known for its high specificity. To address concerns about the specificity, we have now included a Western blot below demonstrating the antibody's specificity. This additional data supports the validity of our findings by confirming that the antibody specifically recognizes nsp15.
2. **Revised Data Presentation:** We repeated the sucrose gradient centrifugation following wild-type IBV infection and confirmed the co-localization of nsp15 with ribosomes. However, we have not yet identified whether nsp15 binds to ribosomal proteins or ribosomal RNA. Therefore, we will temporarily exclude the sucrose gradient centrifugation results and instead focus on the immunoprecipitation of nsp15-RNA complexes using a nsp15 monoclonal antibody to identify the host cell RNAs that bind to nsp15. Additionally, an analysis of nsp15's interactions with host proteins is ongoing. We hope to uncover clues from this analysis that will support further investigation into the host proteins targeted by nsp15, thereby advancing our understanding of its role in regulating host protein translation and antagonizing the host's innate immune response.

High specificity of Nsp15 monoclonal antibody

Nsp15 mono-antibody pull-down samples for RNA sequencing were prepared.

5. Conflicting evidence (Fig. 3). Protein synthesis inhibition phenotypes were evident when over-expressing nsp15 from some coronaviruses (PEDV, TGEV, PDCoV, and SARS-CoV-1) but not others (MERS-CoV and SARS-CoV-2). Theoretically, these data, if true, are hard to understand. The authors do not convincingly explain or reconcile these conflicting experimental outcomes.

**Response:** We appreciate the reviewer's observation regarding the differential effects of nsp15 on protein translation inhibition across various coronaviruses. In both our Western blot and immunofluorescence experiments, we observed that nsp15 from MERS-CoV and SARS-CoV-2 exhibited a weaker inhibition of protein translation compared to other coronaviruses such as PEDV, TGEV, PDCoV, and SARS-CoV-1.

**Addressing the Conflicting Outcomes:**

1. **Variation in Inhibitory Efficiency:** The data suggest that the efficiency of nsp15 in inhibiting protein translation varies among different coronaviruses. This variability could be due to the involvement of other viral proteins that also contribute to protein translation inhibition. For instance, nsp1 from both MERS-CoV and SARS-CoV-2 has been shown to play a significant role in shutting down protein translation. Additionally, nsp14 of SARS-CoV-2 has been reported to contribute to translation inhibition as well.
2. **Role of Other Viral Proteins:** The presence of other viral proteins such as nsp1 and nsp14, which also inhibit protein synthesis, may reduce the reliance on nsp15 for this function in MERS-CoV and SARS-CoV-2. This might explain why nsp15 from these viruses shows a weaker effect on translation inhibition compared to other coronaviruses where these additional mechanisms are less prominent.
3. **Comparative Analysis:** Interestingly, our results indicate that IBV nsp15 plays a particularly strong role in inhibiting protein synthesis and the expression of antiviral genes. In contrast, previous research (e.g., the study "Translational shutdown and evasion of the innate immune response by SARS-CoV-2 NSP14 protein," PNAS, 2021, PMID: 34045361) has shown that in SARS-CoV-2, nsp14 plays a more significant role in translation inhibition than nsp1, nsp5, and nsp15, with nsp15 still contributing to the inhibition to some extent. Conversely, IBV nsp14 does not seem to impact protein synthesis inhibition. The relevant results are shown below.

These findings suggest that while nsp15 has a conserved role in inhibiting protein synthesis across coronaviruses, the extent of its involvement varies depending on the presence and activity of other viral proteins. Different coronaviruses may employ distinct or additional mechanisms to achieve translation inhibition, highlighting the complexity and variability of viral-host interactions. In summary, our results support the idea that although nsp15 contributes to translation inhibition, its role may be overshadowed by other viral proteins in certain coronaviruses. We have revised the manuscript to better reflect these observations and provide a more comprehensive analysis of the potential reasons behind the differences observed.

Below is the sequence similarity of nsp15 from various coronaviruses.

Reviewer #1: (No Response)

Reviewer #2: The mechanism of inhibition of protein synthesis by nsp15 should be investigated. For example, they showed that nsp15 inhibits the expression of proteins in rabbit reticulocyte lysate (Fig. 8). However, it is not clear whether nsp15 inhibits the translation process or just facilitates mRNA decay. They should use Northern blot or qPCR analysis to investigate mRNA stability in this system.

**Response:**

We appreciate the reviewer's suggestion to further investigate the mechanism by which nsp15 inhibits protein synthesis. To address this, we conducted additional experiments:

1. **RNA Immunoprecipitation (RIP):** We used RIP to assess whether nsp15 binds to RNA. Our results confirmed that nsp15 indeed binds to both cellular and viral RNA.
2. **RT-PCR Analysis:** To determine whether nsp15 facilitates mRNA decay, we performed RT-PCR analysis to measure the stability of specific host mRNAs. The results indicated that nsp15 degrades certain host mRNAs, thereby contributing to the inhibition of protein synthesis.

These findings suggest that nsp15 inhibits protein synthesis not only by directly affecting the translation process but also by promoting the degradation of host RNA. The new data supporting these conclusions have been included in the revised manuscript.

The proposed displacement of PABP from the poly(A) tail of mRNA by nsp15 should be tested *in vitro* using recombinant proteins.

**Response:** Thank you for the valuable suggestion. Our study primarily focuses on the dual role of coronavirus nsp15 in inhibiting protein translation by binding to host RNA and preventing the activation of the PKR-eIF2α signaling pathway, thereby reducing the accumulation of viral RNA and mitigating its detrimental effects on viral protein translation.

While we have confirmed that nsp15-induced translation shutdown leads to the nuclear translocation of PABPC1, testing the proposed displacement of PABP from the poly(A) tail of mRNA using in vitro assays with recombinant proteins goes beyond the scope of our current study. However, we recognize the importance of this mechanism and plan to investigate it further in future research.

It is concerning that in contrast to the situation in nsp15 overexpressing cells, there is no nuclear retention of PABPC1 during IBV-WT infection. In addition, the nsp15 EndoU-deficient IBV is capable of inducing the shutoff of host translation. They suggest that this inhibition is solely due to dsRNA-induced eIF2alpha phosphorylation, of which I am not sure. A plausible approach to confirm the existence of eIF2alpha-independent inhibition of host translation by nsp15 is to use ISRIB, which is known to reverse the effects of eIF2alpha phosphorylation (Sidrauski et al. 2015. eLife 4:e05033). If they were right then in the presence of ISRIB the nsp15 EndoU deficient IBV, but not the wild type IBV, would be expected not to inhibit cellular protein synthesis.

**Response:** We appreciate the insightful suggestion. We attempted to use ISRIB to investigate the role of eIF2α phosphorylation in nsp15-mediated inhibition of protein translation. However, we encountered difficulties as ISRIB did not effectively inhibit eIF2α phosphorylation in our system.

To address this concern, we employed an alternative approach by using PKR knockdown to prevent eIF2α phosphorylation. Our findings revealed that in PKR knockdown cells, the EndoU-deficient rIBV-nsp15-H238A lost its ability to inhibit protein translation, whereas wild-type IBV retained this inhibitory effect. These results support our hypothesis that nsp15 plays a dual role in regulating protein translation during viral infection: first, by inhibiting host protein synthesis through a PKR-eIF2α-independent mechanism, likely via targeting host RNA; and second, by cleaving viral RNA to prevent the activation of the PKR-eIF2α-mediated shutdown of protein translation, which would otherwise be detrimental to viral replication.

We have included these findings in the revised manuscript.

Fig. 11. Polysome profiles (A260 gradient traces) should be shown. Also, the interpretation of their western blots is incorrect. eIF4E is known to be mainly present in free protein fractions and not in the mono-ribosome fraction as stated on line 635. Also, Rps6 should be present in the 40S ribosomal subunit fraction in addition to mono-ribosome and polysome fractions. It is also likely that their anti-nsp15 antibody is not sufficiently specific and cross-reacts with a higher molecular weight protein in fractions 10-12 on the right panel. Finally, they are suggested to analyze PABPC1 distribution in gradient fractions.

**Response:** Thank you for your suggestions. We repeated the sucrose gradient centrifugation following wild-type IBV infection and confirmed the co-localization of nsp15 with ribosomes. However, we have not yet identified whether nsp15 binds to ribosomal proteins or ribosomal RNA. Therefore, we will temporarily exclude the sucrose gradient centrifugation results and instead focus on the immunoprecipitation of nsp15-RNA complexes using a nsp15 monoclonal antibody to identify the host cell RNAs that bind to nsp15. Additionally, an analysis of nsp15's interactions with host proteins is ongoing. We hope to uncover clues from this analysis that will support further investigation into the host proteins targeted by nsp15, thereby advancing our understanding of its role in regulating host protein translation and antagonizing the host's innate immune response.

Reviewer #3: 1. CoV nsp15 mutations that disable oligomerization or catalytic activity can no longer inhibit host protein synthesis under the conditions of the experiment; however, host RNA targets of nsp15 catalytic activity are not detected by the experimental approaches used in the study. Host RNA targets of nsp15 should be identified.

Response: Thank you for the valuable feedback. We have addressed this concern by identifying the RNA bound by nsp15 using RNA immunoprecipitation (RIP) experiments. The relevant results, demonstrating that nsp15 targets specific host RNAs, have been incorporated into the revised manuscript.

Reviewer #1: (No Response)

Reviewer #2: Abstract (lines 42-47). I disagree with their statement that "…initiation of viral mRNA translation leads to PABPC1 binding to viral mRNA…" It is well established that PABP binding to mRNA is the cause, not the consequence, of its efficient translation (Kahvejian et al. 2005. Genes Dev., 19:104-113).

**Response:** Thank you for your correction. We have revised the abstract to accurately reflect the established understanding that PABP binding to mRNA facilitates efficient translation, rather than being a consequence of it. The corrected statement has been included in the revised manuscript.

Introduction (line 111). They should mention that poliovirus 2A protease cleaves the initiation factor eIF4G. This cleavage is mainly responsible for the inhibition of cap-dependent translation of host mRNAs (Castelló et al. 2011. J. Biomed. Biotechnol. 2011:369648).

**Response:** Thank you for the suggestion. We have added a mention in the introduction that poliovirus 2A protease cleaves the initiation factor eIF4G, which is primarily responsible for the inhibition of cap-dependent translation of host mRNAs, as reported by Castelló et al. (2011).

Fig 3. Nsp15 of SARS-CoV-2 and MERS-CoV do not inhibit the expression of GFP in contrast to the nsp15 of SARS-CoV-1. Is there a significant structural difference between these proteins?

Response: We are unsure of the structural differences between Nsp15 of SARS-CoV-2 and MERS-CoV compared to Nsp15 encoded by other coronaviruses. Below is the sequence similarity of nsp15 from various coronaviruses.

In both our Western blot and immunofluorescence experiments, we observed that nsp15 from MERS-CoV and SARS-CoV-2 exhibited a weaker inhibition of protein translation compared to other coronaviruses such as PEDV, TGEV, PDCoV, and SARS-CoV-1.

**Addressing the Conflicting Outcomes:**

1. **Variation in Inhibitory Efficiency:** The data suggest that the efficiency of nsp15 in inhibiting protein translation varies among different coronaviruses. This variability could be due to the involvement of other viral proteins that also contribute to protein translation inhibition. For instance, nsp1 from both MERS-CoV and SARS-CoV-2 has been shown to play a significant role in shutting down protein translation. Additionally, nsp14 of SARS-CoV-2 has been reported to contribute to translation inhibition as well.
2. **Role of Other Viral Proteins:** The presence of other viral proteins such as nsp1 and nsp14, which also inhibit protein synthesis, may reduce the reliance on nsp15 for this function in MERS-CoV and SARS-CoV-2. This might explain why nsp15 from these viruses shows a weaker effect on translation inhibition compared to other coronaviruses where these additional mechanisms are less prominent.
3. **Comparative Analysis:** Interestingly, our results indicate that IBV nsp15 plays a particularly strong role in inhibiting protein synthesis and the expression of antiviral genes. In contrast, previous research (e.g., the study "Translational shutdown and evasion of the innate immune response by SARS-CoV-2 NSP14 protein," PNAS, 2021, PMID: 34045361) has shown that in SARS-CoV-2, nsp14 plays a more significant role in translation inhibition than nsp1, nsp5, and nsp15, with nsp15 still contributing to the inhibition to some extent. Conversely, IBV nsp14 does not seem to impact protein synthesis inhibition. The relevant results are shown below.

These findings suggest that while nsp15 has a conserved role in inhibiting protein synthesis across coronaviruses, the extent of its involvement varies depending on the presence and activity of other viral proteins. Different coronaviruses may employ distinct or additional mechanisms to achieve translation inhibition, highlighting the complexity and variability of viral-host interactions. In summary, our results support the idea that although nsp15 contributes to translation inhibition, its role may be overshadowed by other viral proteins in certain coronaviruses. We have revised the manuscript to better reflect these observations and provide a more comprehensive analysis of the potential reasons behind the differences observed.

Fig. 8 shows that nsp15 inhibits the expression of proteins in rabbit reticulocyte lysate. This has nothing to do with nsp15 targeting of nuclear factors as stated in the title.

**Response:** We appreciate your feedback. We acknowledge that Figure 8's results from the rabbit reticulocyte lysate system indicate nsp15's role in inhibiting protein translation in a cell-free environment. This finding highlights that nsp15 can suppress protein synthesis by targeting cytoplasmic factors, not necessarily by affecting nuclear components.

Given this, we revised the manuscript title to "Coronavirus Endoribonuclease nsp15 Induces Host Protein Synthesis Shutdown and Ensures Viral Protein Synthesis by Evading PKR-eIF2α-Mediated Antiviral Translation Shutoff." This new title more accurately reflects the dual roles of nsp15 in both inhibiting host protein synthesis and facilitating viral protein synthesis by evading the PKR-eIF2α-mediated translation shutdown, without implying an effect on nuclear factors.

Discussion (line 732). Did anybody show that stalled translation initiation results in the dissociation of PABPC1 from mRNA?

**Response:** Yes, it has been demonstrated that stalled translation initiation can indeed result in the dissociation of PABPC1 from mRNA. When translation initiation is disrupted, it impairs the normal process of ribosome recruitment and assembly at the mRNA's 5' cap. This disruption can lead to the release of PABPC1 from the poly(A) tail of the mRNA, which is typically associated with the initiation complex. Our results showing sodium arsenite treatment, which induces stalled translation initiation, align with this phenomenon, as we observed the nuclear localization of both PABPC1 and mRNA. This supports the conclusion that stalled translation initiation can cause PABPC1 dissociation from mRNA and its subsequent retention in the nucleus.

Furthermore, mRNA depletion is known to also lead to PABPC1 dissociation from mRNA. PABP accumulates in the nucleus when there is a prolonged reduction in cytoplasmic mRNA levels, whether due to transcriptional inhibition, transient ribonuclease expression, or PABP overexpression.

The nuclear import of PABPC1 appears to occur via the classical importin β karyopherin receptor pathway. Importin α, which binds importin β, interacts with several non-canonical nuclear localization signals in the RNA-recognition motifs of PABPC1. These interactions are outcompeted by poly(A) RNA, suggesting that mRNA-bound PABP is not available for import. Additionally, cycloheximide-induced polysome stabilization has been shown to prevent PABP nuclear localization, indicating that release from translation complexes is a prerequisite for import.

References supporting these findings include:

1. Kumar, G.R., Shum, L., & Glaunsinger, B.A. (2011). Importin alpha-mediated nuclear import of cytoplasmic poly(A) binding protein occurs as a direct consequence of cytoplasmic mRNA depletion. *Molecular and Cellular Biology*, 31, 3113–3125.
2. Afonina, E., Stauber, R., & Pavlakis, G.N. (1998). The human poly(A)-binding protein 1 shuttles between the nucleus and the cytoplasm. *Journal of Biological Chemistry*, 273(21), 13015-21.
3. Gray, N.K., Hrabalkova, L., Scanlon, J.P., & Smith, R.W. (2015). Poly(A)-binding proteins and mRNA localization: who rules the roost? *Biochemical Society Transactions*, 43(6), 1277-84.
4. Burgess, H.M., Richardson, W.A., Anderson, R.C., Salaun, C., Graham, S.V., & Gray, N.K. (2011). Nuclear relocalisation of cytoplasmic poly(A)-binding protein (PABP) 1 and 4 in response to UV irradiation reveals mRNA-dependent export of metazoan PABPs. *Journal of Cell Science*, 124, 3344–3355.
5. Lee, Y.J., & Glaunsinger, B.A. (2009). Aberrant herpesvirus-induced polyadenylation correlates with cellular messenger RNA destruction. *PLoS Biology*, 7, e1000107.

Discussion (line 809). Should read: "…interacts with eukaryotic initiation factors (eIF)…"

**Response:** We have revised the text as suggested to read: "…interacts with eukaryotic initiation factor (eIF)…"

Reviewer #3: Minor points

1. Lines 53, 101, 135, 141, 323, 475, 481, 488, 501, 503, and elsewhere throughout the manuscript. “Host protein translation” is a terrible expression. Host proteins are not translated. Rather, mRNA is translated into protein. Host protein synthesis occurs when ribosomes translate mRNA. Host protein synthesis (is preferred), rather than host protein translation (not ideal).

**Response:** We have revised the manuscript to use "host protein synthesis" instead of "host protein translation" throughout.

2. Line 70, as above. synthesis of polyproteins rather than translation of polyproteins.

**Response:** We have revised the manuscript to use "synthesis of polyproteins" instead of "translation of polyproteins" as suggested.

3. Lines 637, 639. Typo: Rps6.

**Response:** We repeated the sucrose gradient centrifugation following wild-type IBV infection and confirmed the co-localization of nsp15 with ribosomes. However, we have not yet identified whether nsp15 binds to ribosomal proteins or ribosomal RNA. Therefore, we will temporarily exclude the sucrose gradient centrifugation results and instead focus on the immunoprecipitation of nsp15-RNA complexes using a nsp15 monoclonal antibody to identify the host cell RNAs that bind to nsp15. Additionally, an analysis of nsp15's interactions with host proteins is ongoing. We hope to uncover clues from this analysis that will support further investigation into the host proteins targeted by nsp15, thereby advancing our understanding of its role in regulating host protein translation and antagonizing the host's innate immune response.

4. Line 809: eukaryotic initiation factor (eIF).

**Response:** We have corrected "eukaryotic initiation factors (eIF)" to "eukaryotic initiation factor (eIF)" as suggested.
