# Supplementary material for: Coronavirus endoribonuclease nsp15 suppresses host protein synthesis and evades PKR-eIF2α-mediated translation shutoff to ensure viral protein synthesis
Source: PLoS Pathog. 2025 Mar 17;21(3):e1012987. doi: 10.1371/journal.ppat.1012987 (PMC11975131; doi:10.1371/journal.ppat.1012987)
Supplement: Attachment — Submitted filename: Response ro reviewers 20250125.docx [file ppat.1012987.s009.docx]

**Reviewers' Comments:**

Reviewer's Responses to Questions

**Part I - Summary**
Please use this section to discuss strengths/weaknesses of study, novelty/significance, general execution and scholarship.

Reviewer #1: In addition to answering the reviewers' concerns, the author also supplemented RIP-Seq RNA data and GO and KEGG analyses. The paper contains a wealth of data.

Reviewer #2: This resubmitted manuscript explores the role of nsp15 in translational regulation. The authors have made significant experimental efforts to address some of the weaknesses in the originally submitted manuscript, including nsp15 interactome data.

**Part II – Major Issues: Key Experiments Required for Acceptance**
Please use this section to detail the key new experiments or modifications of existing experiments that should be absolutely required to validate study conclusions.

Generally, there should be no more than 3 such required experiments or major modifications for a "Major Revision" recommendation. If more than 3 experiments are necessary to validate the study conclusions, then you are encouraged to recommend "Reject".

Reviewer #1: In the newly added RIP-Seq RNA data, the author mentioned infecting H1299 cells with wild-type viruses. Previous reports have shown that only the Beaudette strain of coronavirus infectious bronchitis virus after long-term domestication can infect H1299 cell. Does the author have any evidence to confirm that the virus has successfully established an infection?

**Response:** Thank you for raising this question. The "wild-type virus" referred to in the manuscript is the IBV Beaudette strain. As you correctly pointed out, only the Beaudette strain, following long-term adaptation, can successfully infect H1299 cells.

In our study, we used the Beaudette strain to infect H1299 cells and subsequently performed immunoprecipitation experiments using a monoclonal antibody against nsp15 to isolate nsp15 and its interactome. To confirm successful infection and validate the immunoprecipitation of nsp15, we conducted Western blot analysis using the nsp15 monoclonal antibody to detect the expression of nsp15. The results clearly demonstrate successful infection of H1299 cells by the Beaudette strain and efficient immunoprecipitation of nsp15. The Western blot data is provided below as evidence.

We hope this clarifies your concern and further substantiates the experimental validity of our approach. Thank you for your valuable feedback.

Figure 13C: H1299 cells were infected with wild-type IBV for 24 hours. Whole-cell lysates were subjected to immunoprecipitation using an anti-nsp15 antibody, with a Mouse IgG antibody serving as the control. Immunoprecipitated proteins were subsequently analyzed by Western blot analysis using the anti-nsp15 antibody.

Figure 14: H1299 cells were infected with wild-type IBV for 24 hours. Whole-cell lysates were subjected to immunoprecipitation using an anti-nsp15 antibody, with a Mouse IgG antibody serving as the control. Immunoprecipitated proteins were subsequently analyzed by Western blot analysis using the anti-nsp15 antibody.

Reviewer #2: The inclusion of interactome data (RNA and protein is very interesting and provides additional support for some of the conclusions), however specific targets that were identified were not explicitly stated, only pathways and GO terms were used. This data would be even more convincing (and of interest to readers) if some discussion of specific targets were included and not simply relegated to the supplementary data file. For example, what were the 6 viral transcripts that were found to interact with nsp15? What does this potentially imply about the role of nsp15 in regulating expression of viral transcripts throughout the replication cycle? Regarding host protein interactions, was PABPC1 found to interact with nsp15? Were there other specific host proteins that were shown to interact with nsp15 that would further support the model proposed? Were some of these interactions validated using IP-WB?

**Response**: Thank you for your insightful comments and for highlighting the importance of explicitly discussing specific targets from the interactome data. We agree that including such details would enhance the impact and clarity of our findings. We provide additional information in the manuscript:

1. Viral Transcripts Interacting with nsp15: In the RIP-Seq analysis, we identified six viral transcripts that interacted with nsp15, including full length genomic RNA/mRNA1 and the subgenomic mRNAs (mRNA2, mRNA3, mRNA4, mRNA5, mRNA6), which encoding the polyprotein 1a and 1ab, structural proteins and accessary proteins S, 3a, 3b, E, M, and N. This interaction implies that nsp15 may play a direct role in modulating the stability, processing, or translation of viral transcripts during the replication cycle. These findings align with our proposed model that nsp15 not only evades host antiviral mechanisms but also fine-tunes viral RNA and the expression of viral genes to optimize replication. We have added this discussion to the revised manuscript for better contextualization.
2. Host Protein Interactions: Among the host proteins identified to interact with nsp15, PABPC1 (poly(A)-binding protein cytoplasmic 1) was indeed found as one of the interactors. PABPC1 plays a critical role in mRNA stability, translation, and turnover, and its interaction with nsp15 supports the hypothesis that nsp15 may modulate host translation machinery to favor viral protein synthesis. In addition to PABPC1, several other host proteins with functions related to RNA processing and translation regulation were identified, rRNA processing proteins, ribosome biogenesis factors, ribosomal components, eukaryotic initiation factors (eIF2S1-3, eIF3A-M, eIF4A1-3, eIF4G1-3), and RNA-binding proteins (e.g., PABPC1, PABPN1, PABPC4, hnRNP, RBM, and others). These interactions suggest a broader role for nsp15 in hijacking host translation pathways. We have expanded the interpret of these findings in the main text, emphasizing their relevance to the proposed model of nsp15 function.
3. **Validation of Interactions:** To validate specific host protein interactions, we performed co-immunoprecipitation followed by Western blot analysis (IP-WB). However, possibly due to the low binding affinity, we were unable to successfully co-precipitate nsp15 and endogenous PABPC1. In future studies, we plan to further investigate how nsp15 targets host proteins to exert its function and to identify its interacting partners more comprehensively.

We appreciate your suggestion to integrate specific targets into the discussion, as this strengthens the conclusions of our study and makes the data more accessible to readers. All relevant details have been incorporated into the revised manuscript and highlighted accordingly. Thank you for your constructive feedback.

**Part III – Minor Issues: Editorial and Data Presentation Modifications**
Please use this section for editorial suggestions as well as relatively minor modifications of existing data that would enhance clarity.

Reviewer #1: (No Response)

Reviewer #2: n/a
